# Robotic automation of droplet microfluidics

**DOI:** 10.1063/5.0064265

**Published:** 2022-02-03

**Authors:** Tuan M. Tran, Samuel C. Kim, Cyrus Modavi, Adam R. Abate

**Affiliations:** 1 Department of Bioengineering and Therapeutic Sciences, University of California San Francisco, San Francisco, California 94158, USA; 2 California Institute for Quantitative Biosciences, San Francisco, California 94158, USA; 3 Chan Zuckerberg Biohub, San Francisco, California 94158, USA

## Abstract

Droplet microfluidics enables powerful analytic capabilities but often requires workflows involving macro- and microfluidic processing steps that are cumbersome to perform manually. Here, we demonstrate the automation of droplet microfluidics with commercial fluid-handling robotics. The workflows incorporate common microfluidic devices including droplet generators, mergers, and sorters and utilize the robot's native capabilities for thermal control, incubation, and plate scanning. The ability to automate microfluidic devices using commercial fluid handling will speed up the integration of these methods into biological workflows.

## I. INTRODUCTION

Droplet microfluidics enables valuable analytic capabilities by performing compartmentalized reactions in emulsions.^1–4^ This has enabled sensitive diagnostics and ultrahigh-throughput screening for digital enzyme-linked immunosorbent assay (dELISA),^5,6^ low-input DNA sequencing with digital droplet multiple displacement amplification (ddMDA),^7,8^ and directed enzyme evolution through single cell analysis.^9^ Other approaches have been commercialized to broadly impact biomedical research, including digital droplet polymerase chain reaction (ddPCR) and single cell transcriptome sequencing.^6,10–13^ A key factor to these commercial successes is the simplicity of their microfluidics that, in a single processing step, compartmentalizes nucleic acids, cells, and barcode beads. More advanced systems perform two microfluidic steps, in which a first emulsion is combined with a second one using a droplet merger device to enable highly multiplexed targeted amplification and single cell genome sequencing (Raindance, Mission Bio).^14–16^ Other applications of droplet microfluidics would be valuable for broad use but are difficult to commercialize due to their complexity. For example, directed enzyme evolution, single cell functional profiling, and multi-omic assays require multiple microfluidic processing steps and incorporate complex modules, like droplet pico-injection and sorting.^3,11,17,18^ While possible in expert labs, manual device operation is prone to error, and the complex workflows are difficult to reduce to a microfluidic chip that can be disseminated for broad use. To increase the impact of droplet microfluidics, strategies for conducting multistep workflows in an automated and reproducible manner are needed.

Robotics afford a route toward reproducibly conducting multistep workflows.^19–22^ Using valves to control fluids, custom interfaces for introducing reagents, and computers to control chip operation, complex microfluidic workflows have been demonstrated^23,24^ for applications in crystallography,^25^ enzyme kinetics,^26,27^ drug screening,^28,29^ and diagnostics.^30^ However, the interfacing and overall control schemes are custom built to the application and cannot easily be extended to others. By contrast, commercial robotic liquid handling is a mature and flexible technology that has been applied to a broad range of biological applications.^31–33^ It is scalable and capable of simultaneous processing of hundreds of samples in well plates, with numerous control and analytical approaches readily available, including thermal and humidity control, reaction purification, chromatography, mass spectrometry, and microscopy.^34–37^

Rather than building custom approaches to automate microfluidic devices, a superior approach would be to leverage the robust and existing infrastructure of robotic fluid handling; this would speed up the integration of droplet microfluidic methods into high throughput labs already invested in automated well plate processing. In the simplest implementations, this has involved integrating microfluidic pumps with commercial single-channel robotic arms.^38,39^ Prior work has also shown that whole liquid-handling robots with multichannel-pipet arms can be interfaced with the channels of continuous-flow microfluidic devices;^40^ for droplet microfluidics, the closest implementation is currently BioRad's QX200 AutoDG, a specialized platform for preparing 96-well ddPCR reaction plates.^41^ To date, a generalizable robotic platform configurable for any droplet microfluidics operation at scale has not been reported.

In this paper, we present robotically automated droplet (RAD) microfluidics, an approach to conduct droplet workflows with commercial fluid handling. The system comprises three components: the fluid-handling robot, microfluidic devices, and an interface for shuttling reagents between them. The system is controlled by a master computer that instructs the robot on which micro- and macro-fluidic operations to perform, which it does without user intervention. To illustrate the approach, we use it to automate the steps of ddPCR and *in vitro* evolution. Our results demonstrate a general strategy by which to automate multistep droplet microfluidic workflows.

## II. MATERIALS AND METHODS

### A. Device fabrication

The microfluidic devices were fabricated using soft lithography. An SU-8 3025 photoresist (MicroChem) was spin coated on a 3-in. silicon wafer, exposed and developed to make a master mold structure. A droplet maker,^13^ a merger,^13^ and a sorter^42,43^ were fabricated to be 20, 60, and 90 *μ*m tall. PDMS elastomer (RTV615, Momentive) was mixed at 10:1 ratio, poured on the master mold, and cured at 65 °C for 1 h. A PDMS slab was removed from the wafer by cutting and the access holes (0.75 mm in diameter) were punched. The channel side of the PDMS slab was plasma-bonded to a glass slide (12-550C, Fisher Scientific) by treating with oxygen plasma for 60 s at 1 mbar (PDC-001, Harrick Plasma). The inner surface of the microchannels was transformed to hydrophobic by flowing in Aquapel®.

### B. Digital droplet PCR

2% HFE oil is composed of HFE (Novec 7500, 3 M) supplemented with 2%(w/w) 008-FluoroSurfactant (RAN Biotechnologies). 5% FC40 oil is FC40 (Sigma) supplemented with 5%(w/w) 008-FluoroSurfactant. 2% HFE oil was used to make a water-in-oil emulsion containing Phusion PCR master mix (M0530S, Thermo Fisher) 0.5 *μ*M of each primer, and 70 fM template DNA (mCherry gene from plasmid pfm301) in the detergent-free buffer (F520L, Thermo Fisher) supplemented with 2.5%(v/v) Tween 20 and 2.5%(v/v) PEG 6000. Flow rates of aqueous and oil phases used for generating droplets were 125 and 250 *μ*l/h, respectively. The oil phase of the collected droplets was exchanged with 5% FC40 oil by instructing the liquid-handling robot to remove oil from the bottom of PCR tubes because the aqueous droplets float on top of the denser fluorinated oil. 5% FC40 oil supports better droplet stability over the course of the PCR thermal cycle. After PCR, the oil phase was exchanged again with 2% HFE oil supplemented with SYBR Green dye (S7563, Thermo Fisher) 1× concentrate to stain the PCR products. The droplets were visualized in a Countess slide (C10228, Invitrogen), which produces a monolayer that is easily imaged on a microscope (EVOS FL, Thermo Fisher) using both transmission and GFP channels. The acquired images were analyzed with the ImageJ software to extract droplet size and fluorescence distributions.

### C. Multistep workflow

1 *μ*M solution of fluorescein isothiocyanate (FITC) labeled dextran (D1845, Thermo Fisher) was automatically loaded into a syringe and emulsified using the same protocol as ddPCR. Then, the FITC droplets were collected into a 96-well plate by the liquid handler. The robot exchanged the oil phase of FITC drops from 2% HFE to 5% FC40. The robotic arm transferred the well plate to the thermocycler, which closed and performed a dummy cycle (no heating or cycling). After the cycle, the thermocycler opened, and the robotic arm moved the well plate back to the pipettor region. The liquid handler performed another oil exchange to revert back to 2% HFE and moved the well plate to automated syringes.

### D. Master scheduler design

**FIG. 1.**
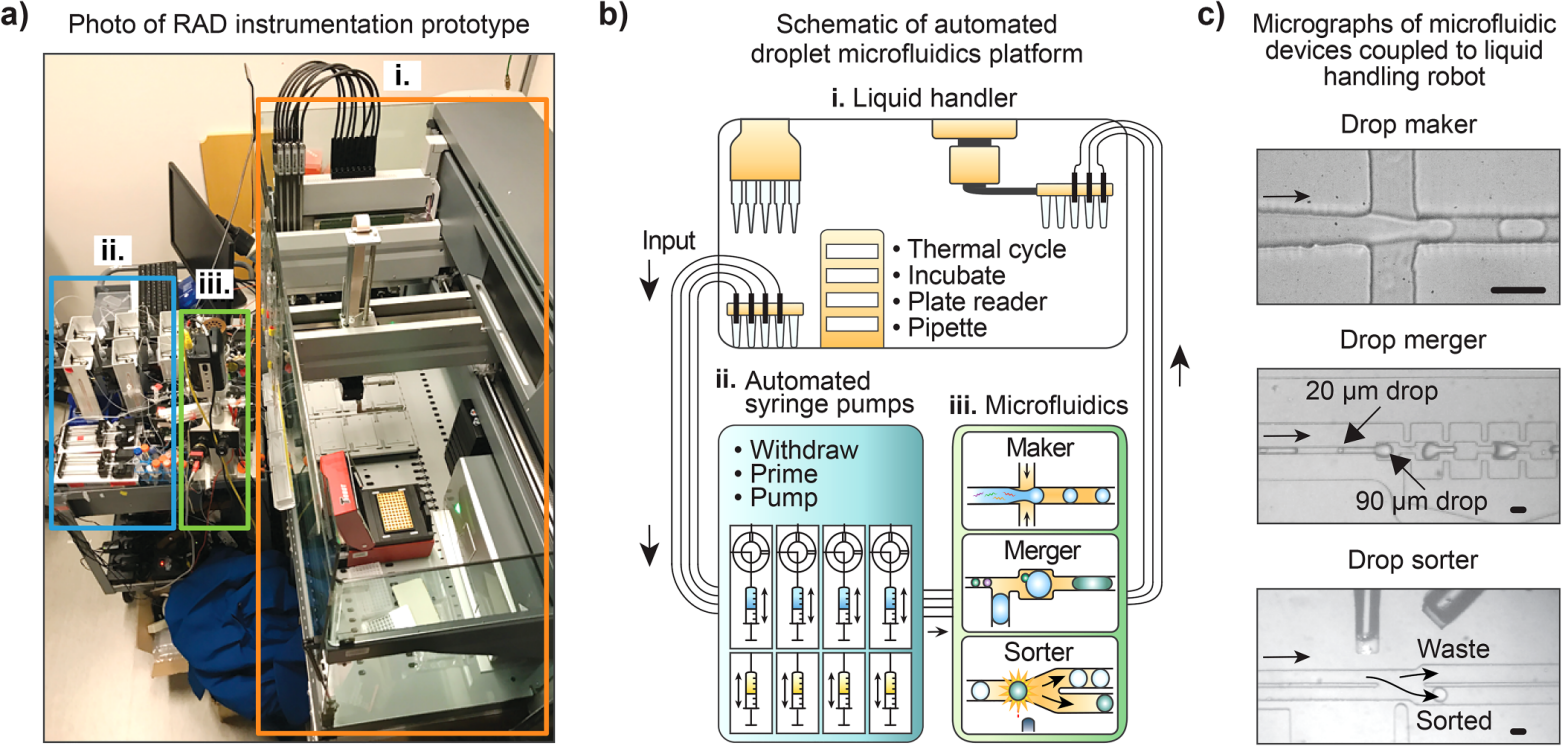
Main components of the RAD microfluidic setup. (a) Instrument consists of three components: (i) Commercial fluid-handling robot capable of processing, incubating, and analyzing fluids; (ii) a fluidic communication highway consisting of arrays of pumps and valves; (iii) a microfluidic breadboard consisting of common modules like droplet generators, picoinjectors, and sorters. (b) Schematic illustrating the instrument flow process. To use the microfluidic devices, the robot loads the requisite reagents into specialized wells connected to the pump array, aspirating and infusing them into the microfluidic devices at controlled flow rates. The entire instrument is controlled by a master computer. (c) Micrographs showing the microfluidic modules operated by the liquid-handling robot. Scale bar is 50 *μ*m.

Automation of droplet microfluidics requires automation of the syringe pumps and liquid handling. An illustration of a modular unit composed of a liquid handler, a pump/valve array, and a microfluidic device. The picture of the entire setup is shown in Fig. 1(a) and Fig. S1 in the supplementary material, with schematics in Fig. 1(b) and Fig. S2 in the supplementary material. A web link to the video recording of the operational steps is shown in Movie S1 in the supplementary material.

1. A master scheduler was written in LabVIEW (National Instruments) to control when the syringe pump or liquid-handling operations are performed.
2. The master scheduler follows a list of steps set by the user.
  a. If it is a syringe pump operation, the scheduler can directly control the syringe pumps to carry out these operations.
  b. If it is a liquid-handling operation, the scheduler simply instructs the liquid handler to perform a specific protocol pre-written in Tecan's EVOware software.
  c. Coordination between the syringe pump operation and liquid handling is performed by creating text files (Fig. S3 in the supplementary material). A text file called “run_fluidics.txt” is created automatically when Tecan is finished with its specific protocol. The LabVIEW scheduler will detect when “run_fluidics.txt” is created and proceed to run the next syringe pump operation. When the syringe pump operation is completed, a text filed called “complete.txt” is created. The creation of this file instructs Tecan to move on to its next pre-written protocol.

### E. Automated syringe pump system

Syringe pumps (NE-501, New Era Pump Systems) were connected to rotary valves from Tecan Cavro pumps (PN 20738707). The Tecan Cavro is capable of pumping but not at the slow flow rates required for droplet microfluidics. The syringe pumps and rotary valves were controlled through custom LabVIEW software. The software rotates the valves to the appropriate position for aspiration or dispensing. It also controls the flow rate and volume dispensed by the syringe pumps.

### F. Fluidic interface between liquid handler and microfluidics

The movement of fluids and emulsions between the liquid handler and microfluidics is facilitated by tubing running between the two systems [Figs. 1(a) and 1(b)]. In all cases, we used polyethylene tubing with an inner diameter of 0.38 mm (BB31695-PE/2, Scientific Commodities Inc.) and minimized the length of tubing between connections. For liquids running from the microfluidic device to the liquid handler, the outlet of the microfluidic device is connected to an appropriately sized tube running to the liquid handler. The open end of the tube is fixed at a position accessible to the liquid handler robot arm carrying a 96-well plate. The robot arm can precisely position any well on the 96-well plate to receive output from the tubing. For fluids running from the liquid handler to the microfluidic device, the open end of the tubing is again positioned at a fixed location on the liquid handler. The other end is connected to the rotary valve of the relevant device. The robot arm can position the 96-well plate such that the tubing hits the bottom of the well. The automated syringe pump can then be instructed to aspirate the contents from the well. For both the aspiration and deposition lines, the tubing was positioned such that it almost touched the bottom of the wells once the robot had finished positioning the plate. For volumes larger than 200 *μ*l, tubes can be directly connected to containers on the platform of the liquid handler and routed to the rotary valves of the syringe pumps. The tubes can be positioned to not interfere with the pipetting motion of the liquid handler into the container.

### G. Liquid handler system

The liquid handler system (Freedom EVO, Tecan) was controlled through the software provided by the manufacturer (Freedom EVOware, build 2.6.17). Protocols for pipetting and liquid transfer are written in the Freedom EVOware software. After each fluidic operation, the robot communication with LabVIEW is achieved by monitoring the text files in a shared network folder.

### H. Optical configuration

A fiber-optic setup was used for detecting fluorescence signals from droplets as previously described.^43^ This optical configuration allowed us to decouple the fluorescence measurement from the microscope. Unlike the conventional epi-fluorescence setup where both excitation and emission light paths share the same objective lens and the photodetector is often attached to the microscope, the fiber-based setup can have the light source and the detector that are physically separate from the microscope. This configuration is useful for monitoring droplet quality over the course of an experiment because the microscope can be used to visualize any part of the operated device while the fluorescence signal is acquired continuously from the separate fiber detection point.

## III. RESULTS AND DISCUSSION

### A. Establishing an automated platform

The RAD microfluidic system consists of separate robotic and microfluidic instruments that fluidically communicate through a modular valve and pump assembly [Figs. 1(a) and 1(b); as detailed in Sec. II]. A master computer controls all components, commanding the robot as needed to process reagents through the microfluidic devices. For fluorescence-activated droplet sorting (FADS), a sorting chip is included on the microfluidic system that isolates select droplets based on user-defined gates. Using pre-programmed operations, the computer directs the liquid-handling robot to load reagents, samples, and oil with surfactant into pump reservoirs; a photograph of the entire rig is shown in Fig. 1(a), with a schematic of the process shown in Fig. 1(b). The key to our approach is the automated syringe handling apparatus that allows us to interface between the robot's wells and the microfluidic devices, such that reagents can be transferred between both systems in either direction. The apparatus consists of syringe pumps, syringe reservoirs, and electronically toggled three way valves. Once well plate loading is complete, the computer instructs the pumps to inject the reagents into a microfluidic chip by reconfiguring rotary valves and pump flows [Fig. 1(b) and Fig. S2 in the supplementary material]. Droplets are generated and processed by the device before traveling back to the robot through tubing and are collected into a reservoir accessible to the robotic pipet.

The transfer process is accomplished by toggling the appropriate valve to transfer into the syringe and then activating the pump in the reverse direction, thereby generating a negative pressure differential at the outlet of the tube connected to the emulsion containing well. This action leads to aspirating the emulsion into the tube and, ultimately, into the syringe. The total volume aspirated is set to be larger than the volume of the emulsion in the well to ensure that the entirety of the emulsion is transferred into the syringe reservoir. For example, for an emulsion volume of 40 *μ*l, an aspirated volume of ∼60 *μ*l is used, which means that about 20 *μ*l of air is also loaded into the syringe. The additional air also ensures that most of the droplets in the connecting tubing are recovered into the syringe since air is immiscible with the carrier oil. The syringe is maintained vertically in the pump [Fig. 1(a)(ii)] such that the dense oil rests at the bottom, the droplets in the middle, and the air at the top. Because air bubbles are not stable in the fluorinated oil, any aspirated air immediately coalesces with the air head above the emulsion in the syringe. After toggling the valve in the other direction to fluidically connect the syringe to the inlet port of the microfluidic device, the syringe pump is then started and the desired volume of emulsion is injected into the device. Because the droplets are less dense than the oil, they are pushed forward by the oil flow into the adjoining tubing and device after the air has been vacated, as in typical droplet reinjection.^42,43^ Syringes that hold emulsion are pre-loaded with 100–200 *μ*l of oil to act as a drive fluid and provide additional volume to cycle droplets through the adjoining tubing, which ensures that essentially all of the emulsion is transferred into the device.

To conduct another microfluidic processing step with a different chip, such as merging or sorting, the robot pipets the recovered droplets into the well reservoir that interfaces with the next chip. Additional processing steps with other chips are conducted in the sequence programmed by the user and can be integrated with processing steps natively supported by the robot, such as environmental control for cell culture or thermocycling for PCR. This setup thus allows the robot and microfluidics to operate as separate systems that can transfer reagents through the pump assembly. This makes the approach modular, allowing the robot to conduct any macro-fluidic processing steps without having to alter the microfluidic system, and similarly the microfluidics to be updated without having to alter the robot or the interface connecting them. This modularity is key to making the approach extended to novel workflows with needs that cannot be easily predicted. To conduct such workflows, the necessary chips are added to the microfluidic component, the robot is reprogrammed as needed for the macro-fluidic processing steps, and the overall operation of both are governed by the master computer. Figure 1(c) shows micrographs of the microfluidic modules integrated with the liquid-handling robot's operations.

**FIG. 2.**
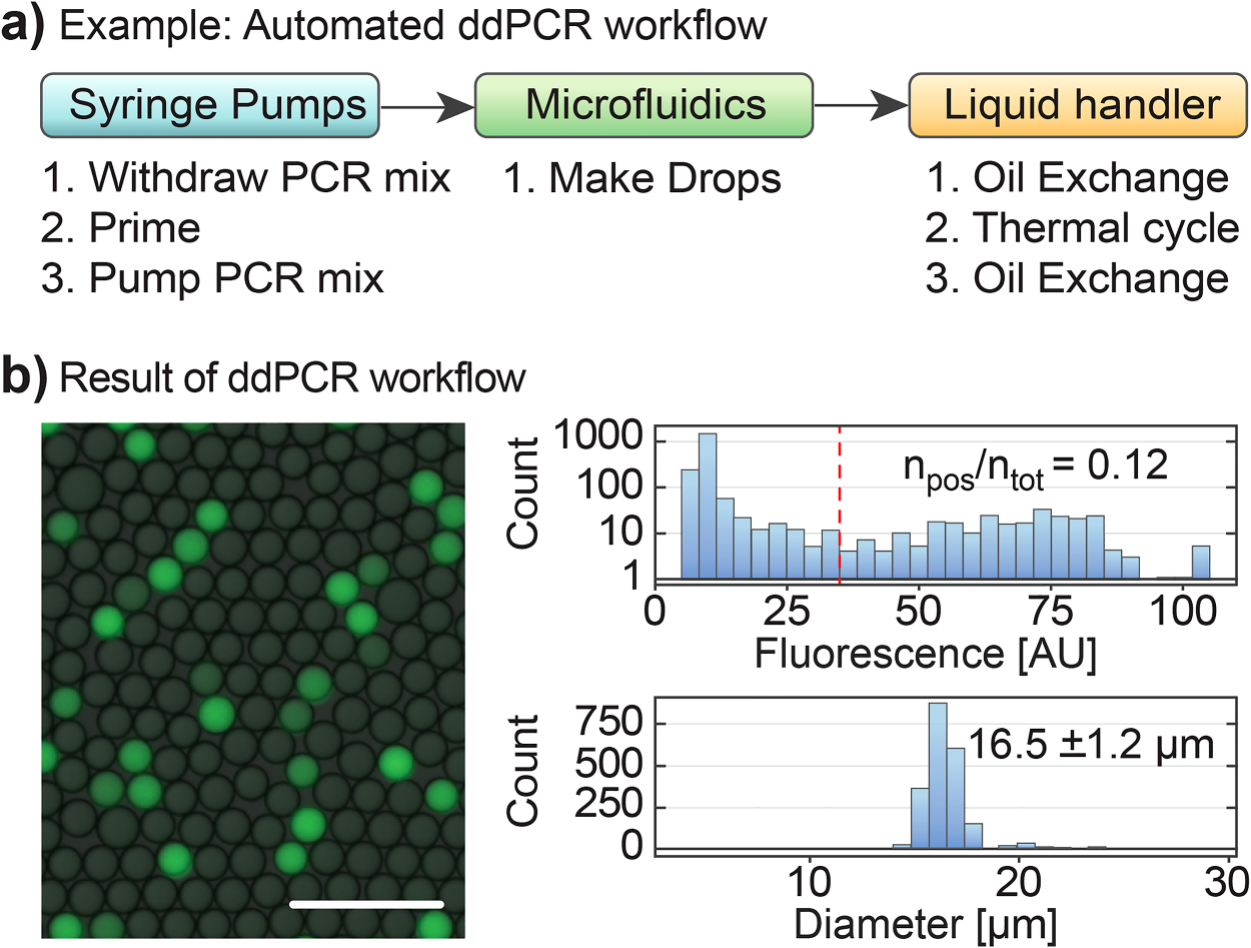
RAD microfluidic automation of ddPCR. (a) The ddPCR workflow consists of three steps, loading of robotically prepared reagents into syringe pumps, encapsulation into monodisperse droplets via flow focusing, and thermocycling of the emulsion aboard the robot. (b) We image samples of the cycled droplets, observing that they are monodisperse and exhibit the characteristic “digital” fluorescence of ddPCR assays. Scale bar is 100 *μ*m.

A common and unavoidable occurrence in almost all droplet microfluidic workflows is droplet coalescence. To minimize it, care must be taken when handling the emulsions, setting the device flow rates, and selecting the best surfactants for stability.^44,45^ A strength of the automation approach is that all emulsion handling is achieved with pumps that can be set to controllably and gently transfer emulsions between reservoirs and devices; consequently, the stability of our emulsions is equivalent to the ones handled manually. To address this, the surfactant and oil carriers can be exchanged [Fig. 2(a)] (Sec. II) between different steps to generate long-term stable emulsions even at the temperatures required for PCR.

### B. Running automated digital droplet PCR

Digital droplet PCR is an important application of droplet microfluidics. Current approaches run on commercial platforms that require separate instruments for droplet generation and reading and require multiple manual processing steps.^10,16^ Consequently, while ddPCR affords major advantages for accuracy and robustness to inhibition,^46^ it remains difficult to utilize in clinical labs due to the challenge of integrating its specialized hardware with the existing robotic infrastructure. Using RAD microfluidics, we automate ddPCR on a commercial robot by supplying it with devices for generating and reading the droplets. In addition to these microfluidic steps, the robot natively supports all other requisite steps, including sample preparation and thermal cycling. The entire workflow, from sample preparation to microfluidic quantitation, is performed by the robot without user intervention. For ddPCR, the robot mixes samples with PCR reagents in plates and then introduces them into a microfluidic droplet generator [Fig. 2(a)]. The robot exchanges HFE oil with thermostable FC40 and surfactant (Sec. II) and loads the emulsion into an onboard PCR machine for thermocycling [Fig. 1(a), red device]. To assess the efficiency of the reaction, we image aliquots of the droplets, observing the characteristic “digital” signal [Fig. 2(b), *left*]. The observed ∼12% positively agrees with the input DNA concentration and droplet loading rate [Fig. 2(b), *upper-right*]. The droplets are uniform in size, exhibiting a diameter variance typical of thermocycled emulsions [∼10%; Fig. 2(b), *bottom-right*]. This shows that RAD microfluidics can perform this important application and affords a strategy to scale ddPCR using commercial fluid handling. The results also show that droplets can be incubated using the same techniques as done by a human, while achieving emulsion handling and stability equivalent to manual workflows.

### C. Running a multi-device workflow

Our system is designed to support arbitrary workflows incorporating numerous microfluidic devices, such as droplet merger^47^ and sorting^42^ [Fig. 1(c), *lower panels*]. To illustrate its flexibility, we thus use the system with updated devices and programming to perform steps common in directed evolution. Directed evolution is valuable in synthetic biology for enhancing an enzyme, pathway, or genetic circuit^48–50^ and is an important application of droplet microfluidics because it benefits from the extreme throughput and ability to analyze single enzyme variants.^51–54^ However, the workflows are complex, requiring iterative cycles of library generation (mutagenesis), enzyme expression (droplet merging), and screening (droplet sorting). For mutagenesis, error-prone PCR can amplify a wild type sequence to generate a diverse library of mutants.^55,56^ Next, each mutant must be tested for activity to identify enhanced variants. This can be accomplished by loading the DNA encoding each mutant into its own droplet containing a cell-free protein expression reagent (CFPRx).^53,57,58^ The mutant sequence is *in vitro* translated to protein and can be incubated with a fluorogenic substrate to generate a signal that quantifies activity.^58^ Because a single DNA molecule is insufficient for reliable protein expression in droplets, to enhance signal, the encoding gene copy number must be increased;^53,58^ this can be accomplished by programming the system to encapsulate and ddPCR-amplify each variant using the protocol described in Fig. 2. The resulting droplets can then be merged with the CFPRx reagent and incubated to express each variant.^53,58^

**FIG. 3.**
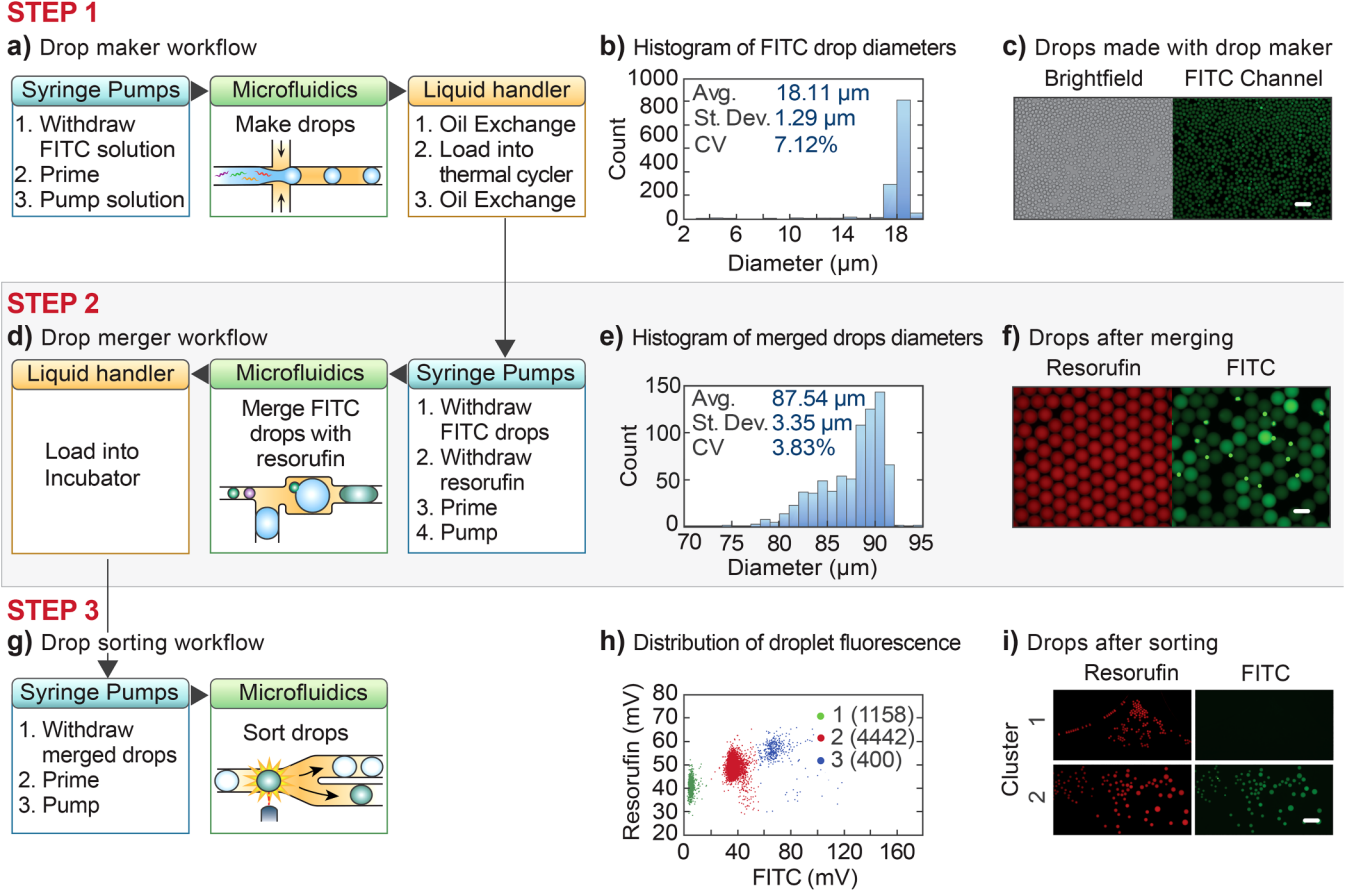
Example RAD automation of a multistep workflow usable for *in vitro* evolution. (a) FITC solution is encapsulated by flow focusing and (b) and (c) the resulting monodispersed droplets are then thermocycled in the PCR machine on the robot. Scale bar is 100 *μ*m. (d) The resulting droplets are merged with CFPRx-containing droplets via the merger device and then incubated. (e) Although the population exhibits polydispersity characteristic of this imperfect process, (f) fluorescence imaging finds a large fraction of the droplets appear to have properly paired and merged. Scale bar is 100 *μ*m. The final population is then (g) sorted via FADS. (h) The pre-sorted droplets exhibit three populations, unmerged, 1:1 merged, and multiply merged, when viewed on a two-color fluorescence scatterplot. (i) By gating specific populations, the instrument recovers droplets with the desired fluorescence properties. Scale bar is 200 *μ*m.

To demonstrate the merging step, we program the robot to first generate FITC containing droplets using a droplet generator and thermocycle them to mimic PCR [Fig. 3(a)]; the droplets are monodispersed, with a coefficient of variation of ∼7% [Figs. 3(b) and 3(c)]. Next, the droplets must be merged with CFPRx, which the robot stains with resorufin to make them distinguishable from the FITC droplets. After thermocycling, the robot then transports the FITC droplets back to the microfluidic system, which reinjects them into a merger device into which the resorufin-stained CFPRx reagent is also introduced [Fig. 3(d)]. The CFPRx reagent is dispersed into droplets using a T-junction, which are paired and merged with the reinjected FITC droplets, generating an emulsion with a coefficient of variation of ∼4% [Fig. 3(e)]. The size distribution indicates that most droplets have been merged, but there is also a subpopulation of smaller, likely unmerged droplets. Thus, while most droplets are FITC-positive, indicating a successful merger, intensity varies across the population, which includes small, unmerged FITC droplets [Fig. 3(f)]. Nevertheless, the resultant emulsion is quite monodispersed and exhibits a merger efficiency equivalent to a manually operated device.

The third step is to recover the target droplets by sorting, which our robot accomplishes via FADS. The robot injects the droplets into the sorter, measures their fluorescence using a fiber-optic detector, and sorts the droplets according to prescribed gates [Fig. 3(g)]. As expected, three populations are detected: cluster 1 with low FITC corresponding to CFPRx droplets that failed to merge; cluster 2 with moderate FITC signal corresponding to desired 1:1 mergers; and cluster 3 corresponding to droplets that have undergone multiple mergers [Fig. 3(h)]. We instruct the system to sequentially sort populations from clusters 1 and 2 into separate wells. Imaging the results [Fig. 3(i)] demonstrates successful recovery and sorting of the two populations with no signs of contamination from the overall process. The observed increase in polydispersity within the collected populations is a common occurrence in sorting processes and our recovered emulsions are equivalent in monodispersity to manually sorted droplets.^42^ It arises from droplets either splitting during sorting or merging post-sorting due to stray electric fields. The lowered monodispersity is generally unimportant since the next step is usually to break the droplets to recover their contents. This shows that the steps required for *in vitro* evolution can be automated using our approach.

## IV. CONCLUSION

A barrier to advancing droplet microfluidics is that workflows are often complex and must be run by an expert. The RAD microfluidics instrumentation breaks down complex workflows into automatable suboperations that are conducted by a commercial fluid-handling robot; no user intervention is required to run a workflow, and different workflows can be run by the system with appropriate programming and choice of devices. These can include most microbiology or biochemistry screening applications,^59,60^ integration with other specialized single cell profiling microfluidic architectures,^61^ multiplexed production of different custom reagent components like functionalized hydrogel beads, or as a platform for scaled testing and characterization of microfluidic module running conditions. This is a powerful advantage because robots can run continuously for extended periods with high reproducibility, which are critical aspects to applying the technology. In addition to making complex workflows simpler and more reproducible, the utilization of commercial liquid-handling robotics provides additional valuable features that normally are not easily built into droplet microfluidic devices, such as reagent purification, thermal incubation, environmentally controlled cell culture, and plate-based fluorescence characterization, among many other commercially available modules.^62,63^ These systems can also leverage the benefits of filtered air workstations and disposable components to reduce the risks of contamination. The biggest barrier to implementing RAD microfluidics is building the pump and valve system that allows interfacing between the robot and microfluidic devices; as of yet, no commercial solution for such an interface exists, and it must be assembled. Though, once set up, the current system can, in principle, operate without any manual intervention. In addition, programming the instrument requires substantial expertise, and the current version does not include fault handling, which will be important for identifying errors and troubleshooting problems. These can be achieved by improved integration of the liquid-handling robot's software to include the code dedicated to microfluidic pump and device functions. Nevertheless, we have shown that this relatively simple interface and controlling scheme allows integration of commercial robotics with droplet microfluidics in a format that should be extendable to a broad variety of workflows and applications.

## SUPPLEMENTARY MATERIAL

See the supplementary material for an enlarged image of the RAD microfluidic system setup (Fig. S1), an expanded schematic of the interfacing between liquid handling and microfluidics (Fig. S2), and a flow diagram detailing how the software is implemented (Fig. S3). A movie of the system in action is also provided as Movie S1.

## Data Availability

The data that support the findings of this study are available within the article and its supplementary material.
